# Supplementary material for: Optimizing Hepatitis C Treatment Monitoring: Is Sustained Virologic Response at 4 Weeks Becoming the New Standard?
Source: Microorganisms. 2024 Oct 10;12(10):2050. doi: 10.3390/microorganisms12102050 (PMC11509943; doi:10.3390/microorganisms12102050)
Supplement: Supplementary file 1 [file microorganisms-12-02050-s001.zip › microorganisms-3242334-supplementary.pdf]

**Supplementary Table S1.** Presentation of laboratory parameters recorded immediately before treatment initiation and after achieving SVR4.

| Variable | Before treatment | After treatment | p |
|---|---|---|---|
| | N=206 | | |
| | Mediana (IQR) | | |
| Leukocytes [$10^9$/L] | 5.74 (6.6 – 8.2) | 6.89 (5.51-8.27) | 0.995 |
| Neutrophils [109/L] | 3.91 (3.1 – 4.75) | 3.45 (2.78-4.37) | 0.582 |
| Hemoglobin [g/L] | 137.0 (124.0 – 155.0) | 140.0 (130.5-158.0) | 0.498 |
| Mean Corpuscular Volume [fL] | 91.5 (87.5 – 94.5) | 92.0 (88.5-95.50) | 0.411 |
| Platelets [109/L] | 201.3 (149.0 – 248.5) | 215.8 (157.0-269.5) | **0.024** |
| Blood glucose level [mmol/L] | 5.2 (4.3 – 6.1) | 5.1 (4.60 – 5.8) | 0.306 |
| Urea [mmol/L] | 5.2 (4.0 – 6.5) | 5.1 (4.1-6.0) | 0.459 |
| Creatinine [μmol/l] | 70.3 (61.0 – 92.0) | 75.0 (64.0-85.5) | 0.523 |
| Sodium [mmol/L] | 137.5 (135.0 – 142.0) | 141.5 (139.0-143.0) | 0.434 |
| Potassium [mmol/L] | 4.2 (4.0 – 4.4) | 4.2 (4.0-4.5) | 0.405 |
| Total bilirubin [mg/dL] | 9.29 (6.5 – 12.4) | 8.1 (6.1-12.2) | **0.001** |
| Direct bilirubin [mg/dL] | 4.2 (3.1 – 6.4) | 3.8 (2.9-5.2) | **0.001** |
| Aspartate Aminotransferase [U/L] | 52.5 (31.0 – 83.0) | 21.0 (17.0-28.0) | **0.001** |
| Alanine Aminotransferase [U/L] | 76.5 (47.0 – 121.5) | 28.0 (21.0-37.0) | **0.001** |
| Gamma-glutamyl transferase [U/L] | 49.0 (30.5 – 84.0) | 29.5 (19.5-42.0) | **0.001** |
| Alkaline Phosphatase [U/L] | 77.5 (61.5 – 99.5) | 72.5 (56.0-92.0) | **0.001** |
| Total proteins [g/L] | 75.5 (71.0 – 78.0) | 75.0 (72.0-79.0) | 0.493 |
| Albumins [g/L] | 38.5 (35.0 – 42.0) | 39.0 (36.0-42.0) | **0.017** |
| International Normalized Ratio | 1.03 (1.01 -1.14) | 1.06 (1.0-1.10) | 0.170 |
| Prothrombin time [sec] | 90.70 (80.0 – 97.0) | 88.5 (79.6-99.0) | 0.378 |
| Fibrinogen [g/L] | 2.45 (2.1 – 2.8) | 2.60 (2.4-2.9) | **0.009** |
| D-dimer [mg/L] | 0.50 (0.27 – 0.85) | 0.50 (0.3-5.6) | 0.620 |
| Alpha-fetoprotein [μg/L] | 3.75 (2.4 – 6.45) | 3.50 (2.1-5.6) | **0.010** |
| Total cholesterol [mmol/L] | 4.14 (3.65 – 4.79) | 4.47 (3.81-5.44) | 0.479 |
| High-density lipoprotein [mmol/L] | 1.1 (1.0 – 1.5) | 1.2 (1.0-1.57) | 0.200 |
| Low-density lipoprotein [mmol/L] | 2.33 (1.8 – 3.0) | 2.36 (1.48-2.36) | 0.090 |
| Triglycerides [mmol/L] | 0.99 (0.79 – 1.43) | 0.94 (0.74-1.35) | 0.319 |

**Supplementary Table S2.** Results of the Cox proportional hazard models: laboratory factors associated with SVR4 i SVR12

| Model 3 | SVR4 | | | | | | SVR12 | | | | | |
|---|---|---|---|---|---|---|---|---|---|---|---|---|
| Variable | Univariante | | | Multivariate | | | Univariante | | | Multivariate | | |
| | HR | 95% CI | P | HR | 95% CI | p | HR | 95% CI | p | HR | 95% CI | p |
| Sex | 0.919 | 0.61-1.23 | 0.401 | | | | 0.836 | 0.48-1.21 | 0.332 | | | |
| Ages | 0.951 | 0.91-0.99 | 0.003 | 0.954 | 0.92-1.04 | 0.120 | 0.921 | 0.89-0.97 | 0.001 | 0.948 | 0.93-1.02 | 0.075 |
| Stage of liver fibrosis | 0.801 | 0.69-0.97 | 0.001 | 0.820 | 0.68-1.12 | 0.195 | 0.884 | 0.75-0.96 | 0.002 | 0.928 | 0.71-1.03 | 0.207 |
| Leukocytes | 1.089 | 0.97-1.13 | 0.141 | | | | 1.106 | 0.99-1.18 | 0.196 | | | |
| Neutrophils | 1.069 | 0.91-1.27 | 0.158 | | | | 1.071 | 0.98-1.16 | 0.365 | | | |
| Hemoglobin | 0.950 | 0.90-1.18 | 0.362 | | | | 0.959 | 0.97-1.08 | 0.412 | | | |
| MCV | 0.906 | 0.84-1.10 | 0.645 | | | | 0.971 | 0.81-1.09 | 0.698 | | | |
| Platelets | 0.912 | 0.82-1.16 | 0.004 | 0.847 | 0.64-1.31 | 0.358 | 0.986 | 0.94-1.03 | 0.256 | | | |
| Blood glucose level | 0.907 | 0.91-1.08 | 0.294 | | | | 0.968 | 0.91-1.07 | 0.698 | | | |
| Urea | 0.259 | 0.81-1.05 | 0.452 | | | | 0.931 | 0.91-1.06 | 0.674 | | | |
| Creatinine | 1.156 | 0.97-1.29 | 0.741 | | | | 1.001 | 0.97-1.01 | 0.180 | | | |
| Sodium | 0.856 | 0.93-1.02 | 0.384 | | | | 0.994 | 0.94-1.03 | 0.651 | | | |
| Potassium | 1.097 | 0.71-1.56 | 0.621 | | | | 1.035 | 0.79-1.45 | 0.697 | | | |
| Total bilirubin | 1.054 | 0.91-1.27 | 0.158 | | | | 1.156 | 0.99-1.21 | 0.383 | | | |
| Direct bilirubin | 0.956 | 0.93-1.03 | 0.094 | | | | 0.997 | 0.98-1.01 | 0.108 | | | |
| AST | 1.064 | 0.97-1.02 | 0.584 | | | | 0.988 | 0.99-1.01 | 0.692 | | | |
| AST | 0.954 | 0.91-1.02 | 0.501 | | | | 0.996 | 0.99-1.01 | 0.479 | | | |
| gGT | 0.932 | 0.99-1.01 | 0.761 | | | | 0.985 | 0.98-1.01 | 0.694 | | | |
| Alkaline Phosphatase | 1.004 | 0.99-1.00 | 0.202 | | | | 1.140 | 0.99-1.20 | 0.654 | | | |
| Total proteins | 1.152 | 0.99-1.24 | 0.362 | | | | 1.039 | 0.99-1.05 | 0.360 | | | |
| Albumins | 0.812 | 0.67-0.99 | 0.034 | 1.098 | 0.73-1.24 | 0.562 | 0.890 | 0.81-0.97 | 0.047 | 0.974 | 0.84-1.051 | 0.543 |
| INR | 0.908 | 0.49-1.60 | 0.652 | | | | 0.848 | 0.51-1.56 | 0.698 | | | |
| Prothrombin time | 0.917 | 0.84-1.10 | 0.096 | | | | 0.979 | 0.99-1.02 | 0.154 | | | |
| Fibrinogen | 1.250 | 0.89-1.36 | 0.497 | | | | 1.049 | 0.94-1.14 | 0.369 | | | |
| D-dimer | 0.987 | 0.82-1.24 | 0.652 | | | | 0.938 | 0.82-1.15 | 0.341 | | | |
| Alpha-fetoprotein | 1.156 | 0.95-1.51 | 0.852 | | | | 1.038 | 0.99-1.01 | 0.947 | | | |
| Total cholesterol | 0.985 | 0.86-1.10 | 0.433 | | | | 0.976 | 0.81-1.16 | 0.630 | | | |
| High-density lipoprotein | 0.920 | 0.64-1.57 | 0.749 | | | | 0.895 | 0.53-1.56 | 0.870 | | | |
| Low-density lipoprotein | 0.907 | 0.69-1.12 | 0.351 | | | | 0.921 | 0.75-1.19 | 0.601 | | | |
| Triglycerides | 0.912 | 0.65-1.43 | 0.760 | | | | 0.821 | 0.65-1.43 | 0.869 | | | |
